# A New Method of Applying Cocain

**Published:** 1900-06-15

**Authors:** 


### A NEW METHOD OF APPLYING COCAIN.

G. Milian, in *Presse Medicale*, 1899, No. 21, page 144, employs for local anesthesia 2 to 4 percent solutions of cocain and ethyl chlorid, sprayed or applied with a cotton plug to the surface to be anesthetized, inducing by this method in five or six minutes sufficient anesthesia for small operations. It does not produce deep anesthesia, but more profound results than the ethyl chlorid alone. It deposits the cocain in the skin or mucous membrane, causing anesthesia of the superficial nerves.—*Merck's Annual*, 1899.

To Control Hemorrhage at Apex of Root after Pulp Removal.—Wet a needle of bibulous paper in bichlorid of mercury and insert in canal. This will control hemorrhage every time.—*J. Y. Crawford, in Alabama Dental Apprentice.*

